# Ecophysiology of *Chloromonas hindakii* sp. nov. (Chlorophyceae), Causing Orange Snow Blooms at Different Light Conditions

**DOI:** 10.3390/microorganisms7100434

**Published:** 2019-10-10

**Authors:** Lenka Procházková, Daniel Remias, Tomáš Řezanka, Linda Nedbalová

**Affiliations:** 1 Department of Ecology, Faculty of Science, Charles University, Viničná 7, 12844 Prague, Czech Republic; 2 School of Engineering, University of Applied Sciences Upper Austria, Stelzhamerstr. 23, 4600 Wels, Austria; 3 The Czech Academy of Sciences, Institute of Microbiology, Vídeňská 1083, 142 20 Prague, Czech Republic

**Keywords:** cryoflora, photosynthesis, cysts, environmental sample, astaxanthin, fatty acids

## Abstract

Slowly melting snowfields in mountain and polar regions are habitats of snow algae. Orange blooms were sampled in three European mountain ranges. The cysts within the blooms morphologically resembled those of *Chloromonas nivalis* (Chlorophyceae). Molecular and morphological traits of field and cultured material showed that they represent a new species, *Chloromonas hindakii* sp. nov. The performance of photosystem II was evaluated by fluorometry. For the first time for a snow alga, cyst stages collected in a wide altitudinal gradient and the laboratory strain were compared. The results showed that cysts were well adapted to medium and high irradiance. Cysts from high light conditions became photoinhibited at three times higher irradiances (600 µmol photons m^−2^ s^−1^) than those from low light conditions, or likewise compared to cultured flagellates. Therefore, the physiologic light preferences reflected the conditions in the original habitat. A high content of polyunsaturated fatty acids (about 60% of total lipids) and the accumulation of the carotenoid astaxanthin was observed. They are regarded as adaptations to cope with extreme environmental conditions of snow that include low temperatures, freeze-thaw cycles, and variable light intensity. The intraspecific ability of adaptation of the photosynthetic apparatus to different irradiance regimes seems to be advantageous for thriving in different snow habitats.

## 1. Introduction

For microalgae, snow and ice surfaces are habitats characterized by a multitude of abiotic stresses, including low nutrients, diurnal freeze-thaw cycles, high UV and photosynthetically active radiation (PAR) at the surface, and an overall short growing season. In these ecosystems, diverse algal communities develop distinct snow discolorations and exhibit individual cellular metabolic profiles [1]. The organisms responsible for such a phenomenon mainly belong to green algae [2,3]. Prominent genera thriving in snow are *Chloromonas* [4] and *Sanguina* (formerly assigned to *Chlamydomonas*) [5].

These microalgae are essential primary producers in such an extreme ecosystem (e.g., [6]), where phototrophic life is restricted to a few specialized organisms. For instance, they provide a basic ecosystem, e.g., for snow bacteria [7], fungi [8], and ciliated protozoa [9]. Snow algae microbial communities play an important role in snow food webs and supply nutrients that are delivered throughout the ecosystem (e.g., supraglacial and periglacial environments) [10].

Insight in complex feedbacks between snow and climate were recently done by [11]. Snow in the mid-latitudes appears to be most sensitive to climate change [12], and mid-latitude areas below 1200 m a.s.l. are assumed to suffer complete snow loss by the end of 21st century [13]. Therefore, snow microbial communities of mid-latitude mountains urgently deserve focused research before their habitats are gone forever.

Organisms living in melting snow have to be well adapted. A key factor for photoautotrophic life is the plasticity of the photosynthetic apparatus, necessary for balancing energy consumption and conversion [14], because light conditions in snow are very variable from surface to deeper layers [15]. At the beginning of the season, cells are deep in the snowpack, thus subject to absence of light. Due to the combination of ongoing melting and upwards migration of flagellates, they become exposed in surface layers to higher light conditions [16]. Thus, constant adjustment of the photosynthetic apparatus is required spatially and temporally in the course of a few weeks. Additionally, snowfields can be quite different in regard of light conditions depending on being shaded, partly shaded under canopy, or permanently exposed at sites without trees. This led to the earlier conclusion that red snow, which is usually fully exposed, comprises species with high light tolerance, whereas green snow, which is usually found at shaded places, is taxonomically different and does not tolerate excessive irradiation [17].

Rapid light curves, acquired either via oxygen turnover or by fluorometry of the plastidal electron transport rates, demonstrate the adaptation of snow algal photosystems (i.e., to low temperatures). This was performed with monospecific blooms like *Chloromonas* (*C.*) *nivalis* [18], *C. polyptera* [19], *C. nivalis* subsp. *tatrae* [20], *Chloromonas brevispina* [21]*, Scotiella cryophila* [22], and *Sanguina nivaloides* (formerly classified as “*Chlamydomonas nivalis*”) [23]. However, these studies used only field samples collected at a certain locality, and to our knowledge, there were no efforts yet to compare either the autecological photophysiology of a single species collected from different mountain ranges, or differently exposed sites in terms of PAR. The first ecophysiological comparison of cryoflora populations from two mountain ranges was done in the case of *Chlainomonas* sp. [16]. However, data on flagellated stages based on laboratory strains or field samples were not available. Thus, the range of light adaptation remained unknown; also the differences between two life cycle stages of typical snow algae in the order Chlamydomonadales (flagellates vs. immotile cysts) were never determined before.

*C. nivalis* (Chodat) Hoham et Mullet was regarded as a cosmopolitan species living in polar and mid-latitude mountainous regions. The taxon was usually identified solely based on the morphology of fusiform cysts bearing elongated cell wall flanges, which usually dominate field blooms. However, molecular studies showed that it comprises multiple taxa, which were recently independently described, e.g., *C. muramotoi* [4].

The cysts used in this study that cause orange snow in mid-latitude mountains in central Europe resembled those of *C. nivalis* as well, but were revealed to be a new taxon of its own by analyses of multiple DNA marker regions. It is described here as *C. hindakii* Procházková & Remias sp. nov., based on molecular and morphologic characteristics of vegetative flagellates and immotile cysts. The aim was not only to describe morphology and ecology, but also to compare the two main life cycle stages in terms of photobiology and fatty acids profiles. Since we were able to find blooms of this species at habitats with quite different conditions of irradiance, we were able to test the hypothesis that populations from high light conditions get photoinhibited at higher irradiances than those from low light conditions. Finally, the carotenoid composition of the reddish cysts was investigated. To sum up, the results of this study are a showcase, helping to understand the geographical distribution, taxonomy, habitat preferences, and intraspecific physiological variability of a new species of snow alga in the snow melting period.

## 2. Materials and Methods

### 2.1. Sampling and Snow Characteristics

**Figure 1:**
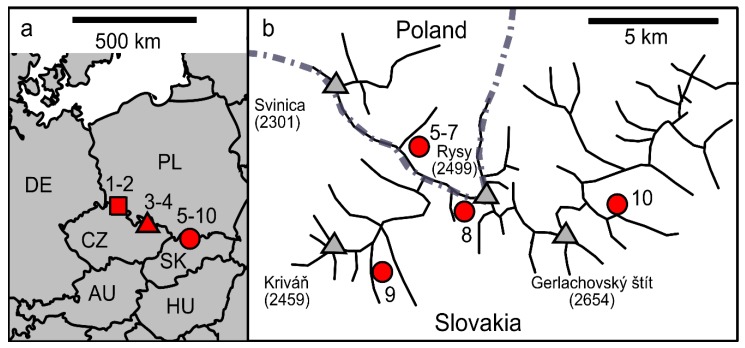
Sampling locations of *Chloromonas hindakii* sp. nov. in (**a**) Krkonoše, Czech Republic (square), Jeseníky, Czech Republic (triangle), and at the High Tatras, Slovakia and Poland (circle), with codes of the European countries (AU, Austria; CZ, Czech Republic; DE, Germany; HU, Hungary; PL, Poland; SK, Slovakia); (**b**) Detailed map of the High Tatras, showing principal peaks with their elevation in meters (triangles).

Orange colored spots of snow were investigated in May and June in the years 2017–2019 in the High Tatra (Slovakia, Poland), Krkonoše, and Jeseníky Mountains (the Czech Republic) (Figure 1, Table 1, Figure S1).

For sampling, the selection of virtually monospecific spots was done with a field microscope according to [5]. Orange snow was harvested with a sterile shovel, placed in 5 l buckets, 1 l thermos bottles, or 50 mL centrifugation tubes, and transported the same day to the laboratory. Prior to photosynthesis measurements, samples were melted gently at darkness overnight at 4–5 °C. Electrical conductivity (EC) and pH of the meltwater were obtained with WTW Instruments (Cond 340i and Inolab, Germany) or with HANNA (Combo EC, ftb Romania). Snow water equivalent (SWE; referred to as ‘snow water content’ in the following reference) was carried out as described previously [20].

### 2.2. Strain Isolation

For obtaining a unialgal strain of *C. hindakii* sp. nov., a subsample of WP129 (containing only sedimented cysts, no flagellates observed) with visible orange coloration was put into sterile 2 mL cryotubes and the meltwater replaced with deionized water. For induction of germination of the cysts, the cells were kept at 1 °C during the day (14 h) resp. −1 °C during the night (10 h) in a Percival LT-36VL (CLF Plant Climatics, Wertingen, Germany). The light intensity generated by fluorescent tubes was approximately 40–70 μmol PAR m^−2^ s^−1^. After several weeks, many cysts developed daughter cells (Figure S2), and subsequently, green flagellates were present in the supernatant; 10 µL aliquots of the latter were transferred into liquid 0.6 N Bold’s Basal Medium (BBM) [24] and irradiation was dimmed to 20–30 μmol PAR m^−2^ s^−1^. In the next step, the culture was used for genetics, microscopy, fluorometry, and lipid analysis. This strain was deposited as CCCryo 531-19 at the Culture Collection of Cryophilic Algae in Germany.

### 2.3. Light and Electron Microscopy

Light microscopy (LM) was performed with an Olympus BX43 at 1000× magnification using oil immersion, equipped with Nomarski Contrast and an Olympus DP27 camera or digital camera DXM 1200F (Nikon, Melville, NY, USA), using cellSens Entry Imaging Software. Scanning and transmission electron microscopy (SEM and TEM) were carried out as described previously [20].

### 2.4. Isolation of DNA, PCR, Sequencing

DNA isolation was carried out with a DNeasy Plant Mini Kit (Qiagen, Germany), as in [20]. If less than 20 mg wet biomass was available, DNA was extracted using the Instagene Matrix Kit (Bio-Rad Laboratories, Hercules, CA, USA) according to [25]. The 18S small subunit ribosomal RNA gene (18S rDNA), internal transcribed spacer region 2 (ITS2 rDNA), and ribulose-1,5-bisphosphate carboxylase/oxygenase large subunit (*rbc*L) marker regions were amplified from DNA isolates by polymerase chain reaction (PCR) using existing primers (Table 2). Amplification reactions were described in [20]. PCR products were purified and sequenced using an Applied Biosystems automated sequencer (ABI 3730xl) at Macrogen Europe (Amsterdam, Netherlands). The obtained sequences of *C. hindakii* sp. nov. were submitted to NCBI Nucleotide sequence database (accession numbers at Table 3).

### 2.5. ITS2 rRNA Secondary Structure Prediction and Phylogenetic Analysis

The methods of annotation and prediction of the secondary structure of the nuclear rDNA ITS2 region were the same as in [20]. The secondary structure of nuclear rDNA ITS2 of *C. hindakii* sp. nov. was drawn using VARNA version 3.9 [32]. The 18S rDNA alignment contained 40 sequences (1567 bp) examined in previous studies [4,22], as well as three specimens of field-collected cysts and *C. hindakii* strain WP129 (= CCCryo 531-19); the *rbc*L matrix consisted of 37 sequences (924 bp), as well as four specimens of field-collected cysts and *C. hindakii* strain WP129 (= CCCryo 531-19); the mesophilic species of the genus *Chloromonas sensu* [33] or the *Chloromonadinia* clade [34] were selected as the outgroup. The best-fit nucleotide substitution model was estimated by jModeltest 2.0.1 [35]. Based on the Akaike Information Criterion, the GTR+I+G model was selected for 18rDNA. Three partitions were set for *rbc*L gene sequences and the following substitution models were applied: TIM1+I+G (first codon position), K80+I (second codon position), and GTR+I+G (third codon position). The 18S rDNA and *rbc*L phylogenetic trees were inferred by Bayesian inference (BI) and maximum likelihood (ML) according to [36], with the minor modification that Markov Chain Monte Carlo runs were carried out for three million generations in BI. Convergence of the two cold chains was checked by the average standard deviation of split frequencies (0.000533 and 0.00105 for 18S rDNA and *rbc*L dataset, respectively). Bootstrap analyses and Bayesian posterior probabilities were performed as described by [36].

### 2.6. Photosynthesis

*In vivo* chlorophyll fluorescence parameters were obtained with a pulse–amplitude modulated fluorometer (PAM 2000, Heinz Walz GmbH, Germany) in a 0.6 mL chamber and cooled with an ice bath to approximately 2 °C. To obtain the relative electron transport rates (rETR) and the light saturation point I_k_, cells were exposed to photon flux densities (PFD) of 5, 9, 34, 67, 104, 201, 366, 622, 984, 1389, 1666, and 2018 µmol photons m^−2^ s^−1^ for 30 s each. Four independent replicates were measured. For further details, see [20].

### 2.7. Pigment Analysis

Carotenoids and chlorophylls were analyzed by HPLC (Agilent 1200 ChemStation) equipped with a quaternary pump and a diode array detector at 450 nm, using a reversed phase YMC C30 Carotenoids column (YMC Europe, Dinslaken, Germany), ID 300 mm × 4.6 mm at 25 °C. α-tocopherol was simultaneously measured with a fluorescence detector (Em/Ex = 295/325 nm). The flow rate was 1 mL min^−1^, analysis time was 20 min following an 8 min post-run prior next injection. The mobiles phases were (A) methanol (gradient grade), (B) methyl *tert*-butyl ether (MTBE; HPLC grade) with 1.3% deionized water (*w*/*w*), and (C) MTBE (HPLC grade). The linear solvent gradient was: From 0 to 4 min at 82% A, 18% B, from 4 to 12 min to 50% A, 0% B, 50% C, from 12 to 20 min to 20% A, 80% C. Post-run was 20% A, 0% B, 80% C from 20 to 23 min, from 23 to 24 min to 82% A, 18% B, and then kept for 4 min. Cells were lyophilized for 48 h and frozen at −25 °C prior use. Extraction was performed with a porcelain mortar and pestle (Z247464 and Z247502, Sigma-Aldrich), using liquid nitrogen for 2 min of precooling and during grinding of cells for 1 min. The cell powder (approximately 10–50 mg) was suspended in approximately 5 mL of chloroform/dichloromethane (2/1) with 1 mM BHT (butylated hydroxytoluene) as antioxidants, the suspension grinded for another minute, and then transferred with a glass pipette into 50 mL plastic tubes for a subsequent post-extraction for at least 12 h at −25 °C. Afterwards, the supernatant was gently evaporated at 30 °C and resuspended in a defined volume of organic solvents (usually 5 mL) composed of 82% mobile phase A and 18% mobile phase B. Prior to injection, the extracts were centrifuged for 10 min at 10,000× *g* and 1 °C. Peak identification was done by peak retention time and peak spectrum in relation to calibration standards (Sigma-Aldrich, Darmstadt, Germany).

### 2.8. Lipid Extraction and Fatty Acid Methyl Esters Analysis (FAMEs) 

The extraction procedure was based on the method of [37], and elution was done from a Sep-Pak Vac Silica cartridge 35cc (Waters; 10 g normal-phase silica) by chloroform (neutral lipids), acetone (glycolipids), and methanol (phospholipids) [38]. All classes of lipids were saponified overnight in 10% KOH in methanol at room temperature. The structures of FAMEs were confirmed by comparison with Gas Chromatography/Mass Spectrometry retention times and fragmentation patterns with those of standard FAMEs (Supelco, Prague) method of [39,40]. Procedures were described in detail by [20].

## 3. Results

### 3.1. Habitat Conditions

**Figure 2:**
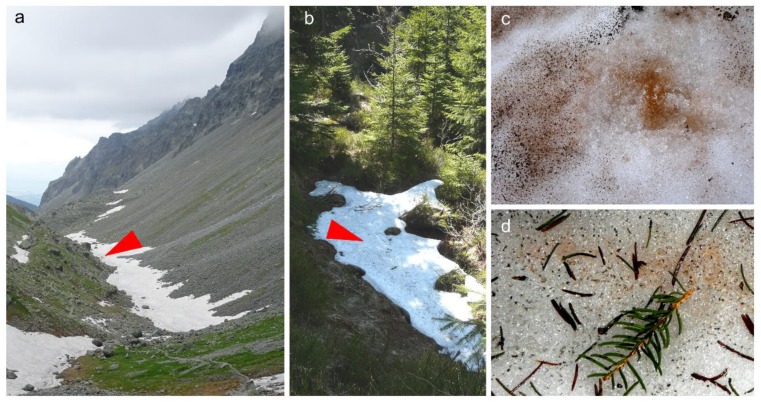
Two representative sampling sites of *Chloromonas hindakii* indicated by red arrowheads and detailed views of the orange snow blooms. (**a**,**c**) Open site above timberline (i.e., high light conditions) in the High Tatra Mountains (sample WP194) and (**b**,**d**) semi-shaded site close to spruce trees (i.e., low light conditions) in the Krkonoše Mountains (sample DD2).

In the High Tatra (Slovakia, Poland), and Krkonoše and Jeseníky Mountains (Czech Republic), orange snow fields were found in May and June 2017 and 2019 at altitudes from 984 to 2082 m a.s.l. (Figure 2, Table 1). The habitat conditions of these localities are shown in Table 4. The orange blooms occurred at open sites above timberline (snow surface: Samples “WP194”, “WP129”, “WP130”, “WP136”; for “LP06”, spots were visible at the surface but the main bloom was harvested at 3–5 cm depth), a semi-shaded site above timberline (close to a boulder, sample “NW”), below dwarf pines (“Rozcestí”). Below timberline, the species was found at open sites (at avalanche slopes, data not shown), semi-shaded (samples “DD2”, “Jes19-1”), and full-shaded sites (“Jes19-6”). For the purpose of rapid light curves measurements, a sample from high light conditions (WP194) and samples from low light conditions (LP06 and DD2) were investigated.

### 3.2. Maximal Population Density and Morphology of Field-Collected Cysts

**Figure 3:**
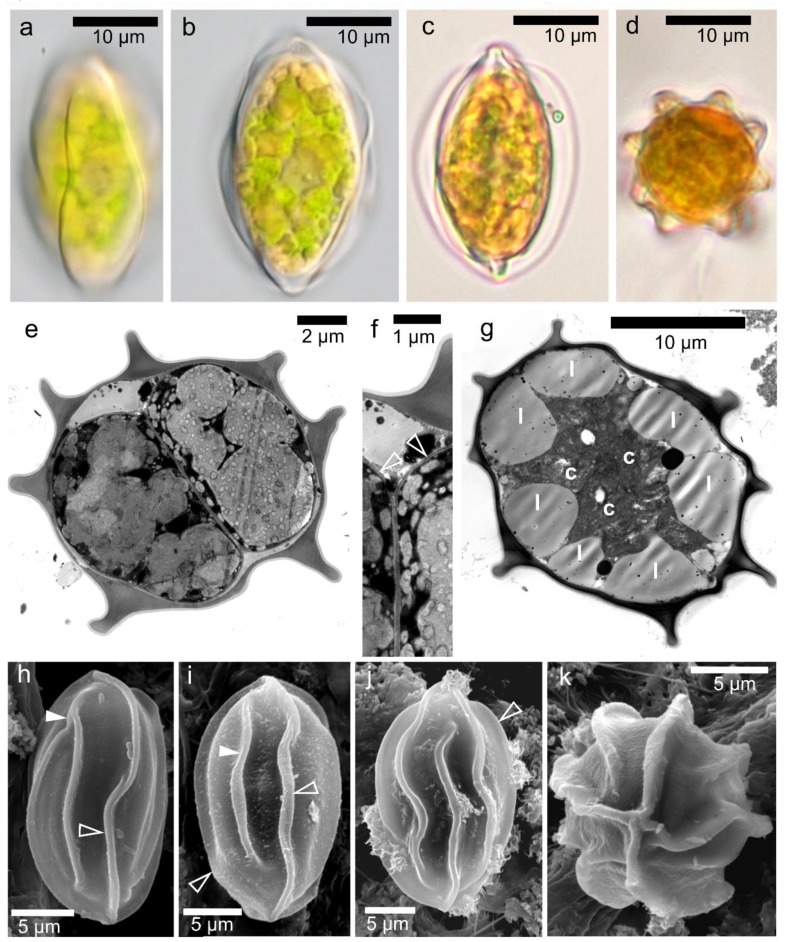
Light and electron microscopy of field-collected cysts of *Chloromonas hindakii* sp. nov.—samples Jes 19-1 (**a**,**b**), WP129 (**c**,**d**), WP194 (**e**,**f**,**h**–**k**), and DD2 (**g**). (**a**–**d**) Light micrographs. (**a**) Surface view showing cell wall flanges. (**b**,**c**) Optical sections, showing cytoplasm containing reddish astaxanthin depots and greenish chloroplast (**b**) and prominent wall flanges reaching the cell apexes (**c**). (**d**) A cell in upright position, showing eight cell wall flanges. (**e**–**g**) Transmission electron micrographs. (**e**) Cell cross-section showing cleavages within the mother cell wall. (**f**) A detailed view of the newly formed cell wall of two daughter cells (empty arrowheads). (**g**) The cytoplasm of mature cysts occupied by large peripheral lipid bodies (*l*) with centrally located chloroplasts (*c*). (**h**–**k**) Scanning electron micrographs, showing characteristic organization of the cell ornamentation. An empty arrowhead indicates a flange reaching from pole to antapex. (**h**) Two flanges joining or a bifurcation of one flange into two independents (white arrowheads). (**i**) An isolated furcated flange (white arrowhead), (**j**) Slightly undulating flanges. (**k**) Apical view of a cell presenting eight flanges in total. Four of them are usually running from apex to antapex.

The population densities reached from 19,950 to 79,100 orange cysts mL^−1^ meltwater (Table 4). Neither green nor orange flagellates were observed in the field material. Cell wall surfaces and intracellular organization were observed by light and electron microscopy (Figure 3). The fusiform cysts with wall surface structures corresponded to those of the aplanozygote of *C. nivalis,* as proposed by [41]. Cells were 18.5–34.3 µm long and 11.9–23.1 µm wide, with a length to width ratio of 1.2:1.9 (Figure S3). Typically, there were 7–10 flanges at the equatorial plane. Wall flanges were either straight or slightly undulating. SEM showed that four prominent flanges always joined at the apex and antapex of the cell, and these flanges ran from one pole to the other. Other wall flanges were shorter and terminated either isolated in the subapex zone, or less often, fused with another flanges. The cytoplasm was dominated by orange–reddish pigmented compartments. Mature cysts had small chloroplast discs (data not shown). The daughter cells within the cysts were smooth-walled (Figure 3e).

### 3.3. Phylogeny and Comparative Analysis of Internal Transcribed Spacer 2

**Figure 4:**
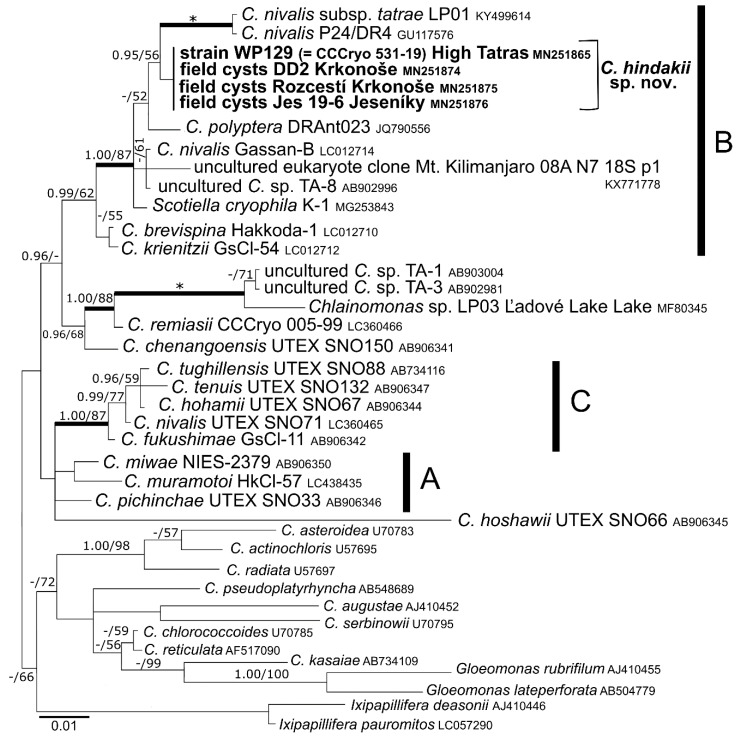
18S ribosomal RNA gene-based Bayesian phylogenetic tree of *Chloromonas* focusing on snow-inhabiting species. *C*. = *Chloromonas*. The labeled clades ‘A’, ‘B’, and ‘C’ correspond to [4]. Posterior probabilities (0.95 or more) and bootstrap values from maximum likelihood analyses (50% or more) are shown. Full statistical support (1.00/100) is marked with an asterisk. Thick branches represent nodes receiving the highest posterior probability support (1.00). Newly obtained sequences are in bold. Accession numbers, strain, or field sample codes are indicated after each species name.

**Figure 5:**
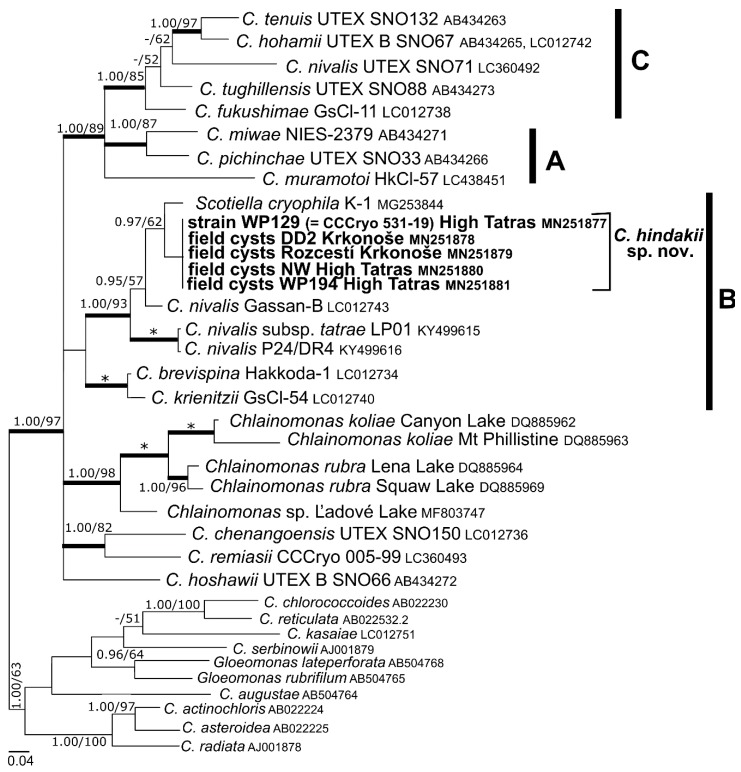
*rbc*L gene-based Bayesian phylogenetic tree of *Chloromonas* focusing on snow-inhabiting species. *C*. = *Chloromonas*. The labeled clades ‘A’, ‘B’, and ‘C’ correspond to [4]. Posterior probabilities (0.95 or more) and bootstrap values from maximum likelihood analyses (50% or more) are shown. Full statistical support (1.00/100) is marked with an asterisk. Thick branches represent nodes receiving the highest posterior probability support (1.00). Newly obtained sequences are in bold. Accession numbers, strain, or field sample codes are indicated after each species name.

**Figure 6:**
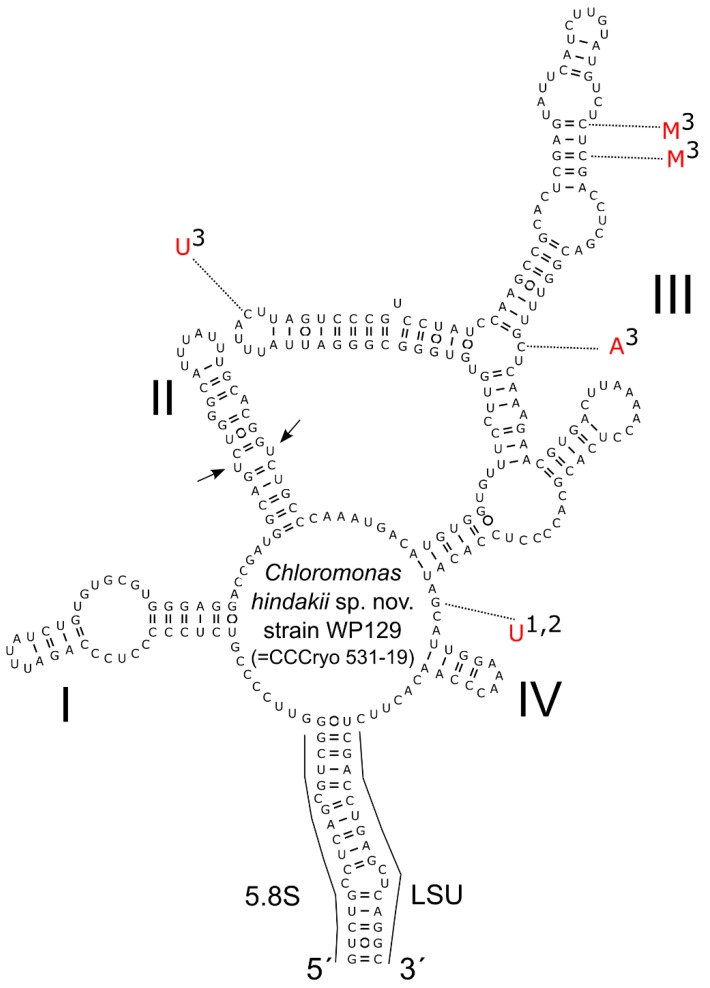
Intraspecific variability in the secondary structure of ITS2 rDNA transcripts of *Chloromonas hindakii* sp. nov.—comparison between the type strain WP129 (= CCCryo 531-19) (accession number MN251865) and field-collected cysts from different localities. The marker was identical for the strain and the following field samples: Rozcestí (MN251867), Jes19-6 (MN251869), LP06 (MN251870), NW (MN251872), WP194 (MN251873). It differed by 1–4 bp in comparison with the field samples (1-DD2, MN251866; 2-Jes19-1, MN251868; 3-WP136, MN251871). Helices are labeled with Latin numbers: I–IV. Nucleotide differences of the field samples are described in red outside the structure and linked by dotted lines. Note the U–U mismatch in helix II (arrows).

The sequences of 18S rDNA and *rbc*L among the strain WP129 (= CCCryo 531-19) and field-collected cysts were identical. According to phylogenies of 18S rDNA (Figure 4) and *rbc*L (Figure 5), this species is a member of “*Chloromonas* clade B” *sensu* [4]. *Chloromonas hindakii* is related to *Chloromonas nivalis* Gassan-B (11 bp different out of 1650 bp in 18S rDNA), *Chloromonas polyptera* DRAnt023 (15 bp different out of 1653 bp in 18S rDNA), *Scotiella cryophila* K-1 (21 bp out of 1680 bp in 18S rDNA), and *Chloromonas nivalis* subsp. *tatrae* LP01 (34 bp out of 1683 bp in 18S rDNA). The new species formed a well supported subclade with other specimens of field cysts identified as *Chloromonas nivalis* P24/DR4 from the Austrian Alps and *Chloromonas nivalis* subsp. *tatrae* from the High Tatra Mountains (Slovakia) in 18S rDNA phylogeny. In contrast, the new species formed a well supported subclade together with *Scotiella cryophila* K-1 from the Austrian Alps in the *rbc*L phylogeny. Within all investigated samples (Table 3), the number of nucleotide differences in the entire ITS2 region ranged from 0 to 4 bp, and no compensatory base changes (CBCs) were detected (Figure 6). Conversely, one CBC was found between strain WP129 (= CCCryo 531-19) and an uncultured environmental clone ALBC6 from Switzerland (Figure S4), and between strain WP129 (= CCCryo 531-19) and *Chloromonas nivalis* Gassan-B from Japan (Figure S5), even near to the 5’ apex of III helix, the most conserved part of ITS2 [42].

### 3.4. Morphology of Vegetative Cells of *Chloromonas hindakii* sp. nov.

**Figure 7:**
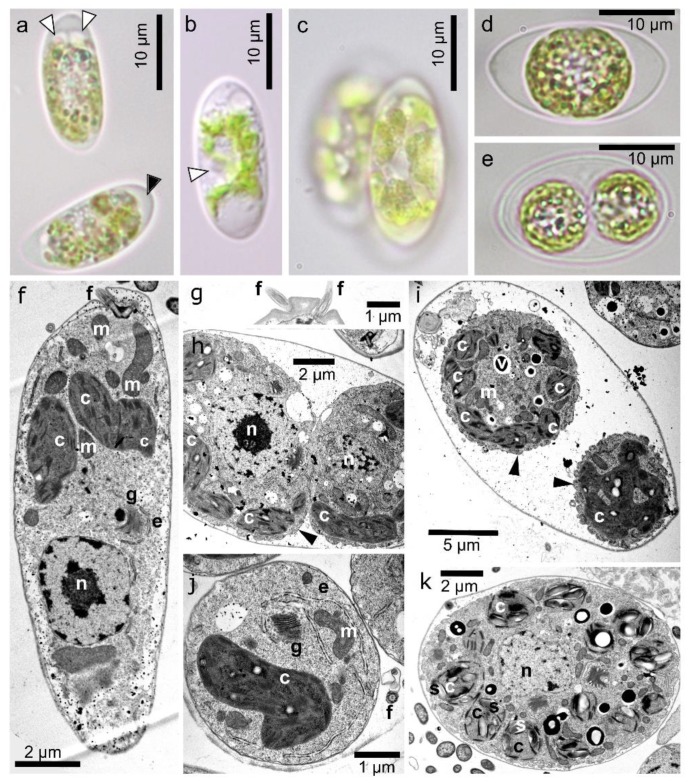
LM and TEM of vegetative cells of *Chloromonas hindakii* sp. nov. strain WP129 (= CCCryo 531-19): (**a**–**e**) LM. (**a**) Optical section of the cell, showing two contractile vacuoles (white arrowheads) and a chloroplast not totally occupying the posterior end of the protoplast (black arrowhead) within the cell. (**b**) Position of the nucleus (white arrowhead) within the cell. (**c**) Sporangium and plate-like sections of the plastid. (**d**,**e**) Development of two spherical daughter cells. (**f**–**k**) TEM. The following structures are labeled: Undulating plasmatic membrane (arrowhead), chloroplast (*c*), endoplasmic reticulum (*e*), flagellum (*f*), Golgi body (*g*), mitochondrion (*m*), nucleus (*n*), starch (*s*), vacuole with crystalline content (*v*). (**f**) Longitudinal cell section, showing position of nucleus. (**g**) Papilla shape. (**h**) Cell cleavage. (**i**) Two spherical daughter cells in mother cell wall, still lacking own cell wall. (**j**) Detail of one out of four spherical daughter cells with newly developed cell wall, still inside the sporangium wall. Note prominent rough endoplasmic reticulum. (**k**) Transversal cell section of an older cell with many discoid chloroplasts including starch grains.

The strain consisted of green and solitary cells, bean-shaped with a rounded posterior end, 7–12.5 μm wide and 17–24.5 μm long (Figure 7). Cells had two equal flagella (if present) at the anterior end, a single chloroplast not fully occupying the posterior end of the protoplast (Figure 7a), two contractile vacuoles near the base of the flagella (Figure 7a), and the wall had no prominent anterior papilla (Figure 7b). The compact, elongated to slightly bean-shaped chloroplast had an emargination in the median region where the nucleus is usually located. The plastid was further lacking an eyespot and pyrenoids. The nucleus was almost spherical, located in the ventral half in the middle of the protoplast (Figure 7b). Asexual reproduction occurred via zoospore (autospore) formation. Generally, two, four, or rarely, eight daughter cells were produced within the parental wall (Figure 7c–e). Sexual reproduction was not observed in the culture, even under nitrogen starvation; nor was the formation of cysts or accumulation of secondary reddish pigments. For testing temperature preferences, cells of *C. hindakii* were exposed to 15 °C but started to decay after 2 weeks, whereas at 5 °C they grew well long-term, and likewise at 1 °C (data not shown).

Figure 7f shows an overview of a flagellate by TEM. The papilla was small and flattened (Figure 7g). Cytokinesis took place in equatorial position of the cell (Figure 7h), resulting either in two spherical cells surrounded by undulating cytoplasmatic membrane only (Figure 7i), or resulting in two elongated cells per sporangium with a prominent rough endoplasmic reticulum (indicating active metabolism). The daughter cells already possessed flagella (see an ultrastructure trait marked with “f”) (Transversal section, Figure 7j). Prior the release from the mother cell, the flagellates developed their own cell wall. The majority released flagellates were bean-shaped, but unusual spherical cells were also observed. Older, mature flagellates contained numerous small discoid chloroplasts with starch grains (Figure 7k).

### 3.5. Taxonomic Treatment

***Chloromonas hindakii* Procházková & Remias sp. nov. (Figure 3 and Figure 7)**

DESCRIPTION: Vegetative cells solitary, having two equal flagella, two contractile vacuoles near the base of the flagella, a single chloroplast, single spherical nucleus positioned in the ventral half at the middle of the cell, without prominent anterior papilla. Cells elongate, kidney-shaped or bean-shaped with rounded posterior end; 7–11.5 µm wide and 17–24.5 µm long. A single chloroplast not occupying the posterior end of the protoplast and lacking eyespot and pyrenoid. Asexual reproduction by formation of two, four, or eight zoospores within the parental cell. Cell aggregates not observed in old cultures. Sexual reproduction unknown. Zygotes or cysts elongate to fusiform, 18.5–34.3 µm long and 11.9–23.1 µm wide, with length to width ratio within a range of 1.2–1.9. Cell walls with rib-like surface structures, such flanges are either straight or slightly undulating. Four prominent flanges always join at the apex and antapex of the cell, and they run entirely from one pole to the other. Further wall flanges are shorter, i.e., terminating either isolated at the subapical zone or less often fusing with another flange, or representing solitary flanges. The cytoplasm of cysts or zygotes usually contains reddish carotenoid droplets. The species differs from any other described representatives of the genus *Chloromonas* in the nuclear 18S rDNA, ITS rDNA, and plastid *rbc*L gene sequences (accession numbers: MN251865, MN251865, and MN251877, respectively).

HOLOTYPE: Specimen WP129 deposited at the Culture Collection of Algae of Charles University in Prague (CAUP), Czech Republic; material consists of resin-embedded vegetative cells from culture strain WP129.

AUTHENTIC STRAIN: WP129. The strain was deposited at the Culture Collection of Cryophilic Algae (CCCryo, Available online: http://cccryo.fraunhofer.de/web/strains) in Potsdam, Germany, as a living culture, strain number CCCryo 531-19.

TYPE LOCALITY: N49°11.424 E20°03.153, snowfield at Dolina za Mnichem, High Tatra Mountains, Powiat tatrzański, Bukowina Tatrzańska, Lesser Poland, Poland.

OTHER DISTRIBUTIONS: High Tatra Mountains (Slovakia), and Krkonoše and Jeseníky Mountains (Czech Republic).

ETYMOLOGY: The species epithet ‘*hindakii*’ is *in memoriam* to Prof. František Hindák (1937–2019), a Slovak phycologist with influential contributions on microscopic algae. He described many new genera and species, including snow algae (e.g., [43,44]), and was one of the pioneers in successfully cultivating snow algae at laboratory conditions [45].

### 3.6. Photosynthesis

**Figure 8:**
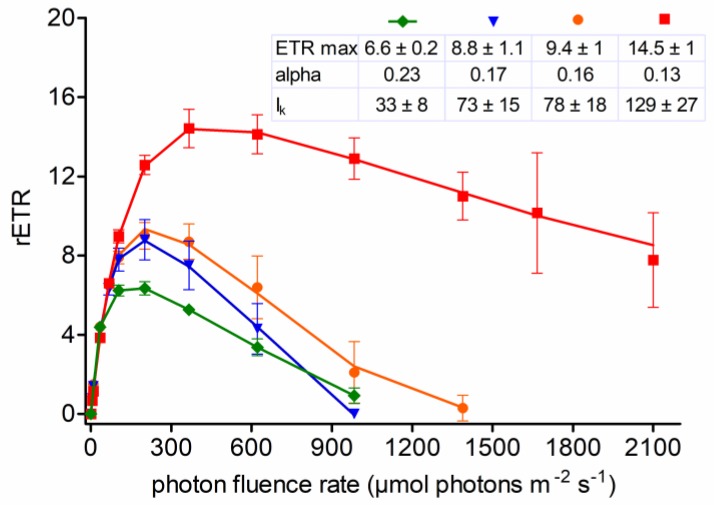
Rapid light curves showing the intraspecific photosynthetic flexibility of *Chloromonas hindakii* sp. nov. The effect of increasing photon fluence rates (x-axis) on the relative electron transport rate (rETR; y-axis) of chloroplasts was measured for vegetative flagellates (green diamonds, strain WP129 (= CCCryo 531-19) grown in the lab) and non-motile field-collected cysts (blue triangles—semi-shaded site close to a spruce canopy, sample DD2; orange circles—site above timberline, population located mainly 3–5 cm below snow surface, sample LP06; red boxes—site above timberline, population at snow surface, sample WP194). Values are means of four replicate measurements (*n* = 4, ± SD). The data points were fitted to the photoinhibition model of [46].

The photosynthetic activity of *C. hindakii* sampled at locations with different light conditions was tested and rapid light curves generated (Figure 8). Additionally, the performance of field cysts was compared with the laboratorial strain. Generally, the cysts were not dormant in terms of photosynthesis, as indicated by rETR. For the strain, and field cysts from low light conditions (below dwarf pine or 5 cm below the snow surface at a site above timberline), photoinhibition occurred above 200 μmol PAR m^−2^ s^−1^. The strain showed an α value of 0.23, a relative ETRmax of 6.6 ± 2.3, and an I_k_ value of 33 ± 8 μmol PAR m^−2^ s^−1^. Field cysts from low light conditions had one-quarter lower alpha values (0.17 and 0.16) and two-fold higher I_k_ (73 and 78). In contrast, cysts from the snow surface above timberline (i.e., high light conditions) showed later signs of photoinhibition, starting from three times higher irradiances (600 μmol photons m^−2^ s^−1^); they also showed a two-fold higher ETRmax (14.5 ± 1) and four times higher I_k_ (129 ± 27), but a nearly twice lower α (0.13), when compared to the strain.

### 3.7. Pigment Composition

The orange pigmentation of cysts of *C. hindakii* was caused by the secondary (non-plastid) carotenoid astaxanthin. Its abundance and those of other pigments and α-tocopherol was calculated in reference to chlorophyll-*a* (Table 5). Astaxanthin comprised 19.8% and 22.1% of all pigments, respectively (WP194, LP06). At HPLC, it occurred in a range of several peaks with identical absorption spectra, all of them likely esters with different fatty acids (Figure S6). Chlorophylls (*a* and *b*) comprised 57.7% and 57.6% of all pigments, primary (plastid) carotenoids represented 22.5% and 20.3% of the pigment pool. The overall ratio of cysts for astaxanthin to chl-*a* was 0.4 and 0.5, respectively. Further details are given in Table 5. In contrast, the laboratorial strain always stayed green (data not shown).

### 3.8. Fatty Acid (FA) Composition

The relative content of FAs (percentage of total lipids and percentage of the three major lipid groups) in *C. hindakii* is given in Table 6. FAs with 14 to 18 C prevailed in field cysts and the strain (WP129). Cells showed high levels of PUFAs (65.8% and 58.1%, respectively, of total lipids), whereas the content of saturated acids (SAFA) did not exceed 23% (mainly palmitic acid, 16:0). The main monounsaturated fatty acid (MUFA) was oleic acid (18:1 (9Z), 9.8 ± 3.6% and 9.4%). The major PUFAs were identical for field cysts and the strain flagellates but differed in relative proportions. The dominant PUFA was α-linolenic acid (18:3 (9Z,12Z,15Z), 25.9 ± 3.7% and 31.6%, respectively). Flagellates had three times higher content of hexadecatetraenoic acid (16:4 (4Z,7Z,11Z,13Z), 28.5 ± 4.6%) than cysts (10.3%) and similar levels of steariadonic acid (18:4 (6Z,9Z,12Z,15Z), 5.9 ± 0.2 and 4.7%). Only minor differences in the FA composition between the three lipid classes of vegetative flagellates were observed (Table 6). PUFAs dominated the neutral lipids, phospholipids, and glycolipids (63.8%, 65.1%, and 75%, respectively). α-linolenic acid was up to twice higher in phospholipids than in neutral and glycolipids.

## 4. Discussion

### 4.1. Geographic Distribution and Ecology

Based on the discoveries in several mountain ranges of Poland, Slovakia, and the Czech Republic, *C. hindakii* sp. nov. seems to be a common member of the local cryoflora. Remarkably, it causes orange blooms both below and above timberline, contrary to many other snow algae, which usually thrive either in exposed or shaded habitats. The distribution of the species, at altitudinal gradient, ranged from montane to subalpine and alpine vegetation belt; e.g., for Krkonoše Mountains, see [47] (p. 271). The discoloration may vary; along with lower population densities, the snow was once reported to be pinkish [48]. A further, hitherto unidentified snow alga with eyespot and prominent cellular tail from the same sampling region reported as ´*C. nivalis*´ [48] is not in accordance to the morphology of *C. hindakii* (compare Figure 2b,c in [48] to Figure 7a,b in this study). However, in the study of [48], cysts morphologically identical to *C. hindakii* were found in Krkonoše at a wide altitudinal gradient from 740 to 1545 m a.s.l., at shaded (broad leaf and spruce trees) as well as at open sites (assigned to *C. nivalis*, see Figure 2e–g in [48]). Moreover, *C. hindakii* may occur also in other mountains in Slovakia [49]. Moreover, it may be found in the Bulgarian Vitosha Mountains, since [50] reported morphologically corresponding cysts in term of cell size, prominent cell apexes, and number and organization of longitudinal cell wall flanges. The overall geographic distribution of *C. hindakii,* including further regions like the European Alps, is not known yet, but distinct morphologic traits reported in this study and deposited molecular marker sequences should help for a future correct recognition.

### 4.2. Taxonomy and Related Species

To our knowledge, the vegetative strain of *C. hindakii* is the first available with a “*C. nivalis*-like” morphology of cysts (fusiform cells with prominent cell wall flanges) within the *Chloromonas* ‘snow clade B’ (*sensu* [4]). In many cases for snow algae, cysts were not made to germinate for generating a flagellate culture. Comparative analysis of the ITS2 rRNA secondary structure showed that *C. hindakii* is closely genetically related to an uncultured environmental clone, ALBC6, from a glacier forefield in Switzerland (accession number JX435348, [51]) and to *C. nivalis* Gassan-B from snow in Japan (accession number LC012758, [52]), indicating that the closest relatives also thrive in cold habitats. However, by using solely light microscopy, the cysts of *C. hindakii* have probably been reported several times as those of *C. nivalis* (as “*Scotiella nivalis*”) in the Krkonoše [48,53,54,55,56] and High Tatra Mountains [49,57,58,59,60]. On the other hand, this is the first report about cryoflora from the Jeseníky Mountains. By the help of electron microscopy and molecular methods, it was possible to describe this new species out of the “collective” taxon *C. nivalis*. This goes along with the description of further new snow algae like *C. hoshawii* or *C. remiasii*, which were formerly assigned to *C. nivalis* as well [61].

### 4.3. Morphology of the Field Cysts

Field-collected cysts in snow identifiable by LM as the cosmopolitan taxon *C. nivalis* were recently shown to represent multiple species [61]. Detailed morphological comparisons of the cyst (i.e., cell wall flanges distribution and characterization [20]) and flagellates [61] is crucial for classical species determination. Morphologically, cysts of *C. hindakii* differ from its close molecular relative *Scotiella cryophila* K-1 in having significantly less elongated cysts (the latter has length to width ratios from 2.1 to 4.7 [22]). Moreover, *C. hindakii* cysts differ from genetically distinct *C. nivalis* in the Austrian Alps [18], and *C. nivalis* subsp. *tatrae* [20] in the High Tatras in their cell size (larger cysts) and more prominent cell wall flanges. The number of cell wall flanges when observed at equatorial position is lower for the new species compared to *C. nivalis* subsp. *tatrae* (the latter has (9)10 to 12(14) flanges). These two species co-occurred occasionally in the High Tatra Mountains (own observations). Aplanozygotes of *C. nivalis* from North America [41] possess a lower number of flanges at cross-section (six to eight), are similarly large (16–37 μm × 10–27 μm), but lack fusing/diverting flanges apart from the apex; however, here are no sequences available for these cysts yet. In addition, the identity of several vegetative *Chloromonas* strains isolated from the collection sites of *C. nivalis* in North America [41] is still unknown, and morphological details of their source material are missing (i.e., UTEX SNO16, UTEX SNO17, UTEX SNO18, UTEX SNO19, and UTEX SNO21). Matsuzaki et al. [4] reported for *C. muramotoi* a regular cell wall flange organization (i.e., four long flanges reached to both poles of the cell) similar to *C. hindakii*; however, *C. muramotoi* can be discriminated by its markedly smaller cell sizes (9.1–13.4 μm wide and 15.6–22.4 μm long). Moreover, a bifurcation of wall flanges was not observed for *C. muramotoi* and shorter flanges were only medially located (i.e., not reaching subapical cell regions). In this aspect, the new species is somehow similar to another snow alga, *C. miwae*, which, however, is smaller (10–16 μm wide and 20–26 μm long) and lacks visible accumulation of secondary carotenoids [52]. Mature cysts of *C. hindakii* have small chloroplast discs like all other cysts of *Chloromonas* species dwelling in snow (e.g., see chloroplast shape in Figure 22 in [20]). To conclude, it is generally possible to distinguish the cysts of *C. hindakii* morphologically from cysts of other snow algae.

### 4.4. Morphology of the Strain and Context to C. nivalis

The vegetative flagellates of *C. hindakii* differ from the five other most similar looking elongate to bean-shaped *Chloromonas* species isolated from snow as follows: The chloroplast of *C. hindakii* does not occupy the posterior ending of the protoplast, in contrast to *C. krienitzii* [52]. *C. hindakii* is larger in size than *C. fukushimae* when kept at the same cultivation conditions (4–9 µm × 15–23 µm; [62]) and develops only rarely eight zoospores in zoosporangia. Large cell aggregates were not observed in old cultures of *Chloromonas hindakii* in opposition to *Chloromonas polyptera*, where aggregates are made up of more than 16 cells [63]. *Chloromonas hindakii* differs in chloroplast morphology from *C. rostafinskii*—the latter has a cup-shaped chloroplast [64]. *Chloromonas hindakii* differs from *Chloromonas rubroleosa* in papilla shape and number of contractile vacuoles near the base of flagella—the latter has prominent bimamillate papilla and four contractile vacuoles [65]. Additionally, the vegetative morphology of *C. hindakii* is different from species not possessing bean-shaped flagellates, such as the North American strain UTEX SNO71 [4], which was recently proposed to represent the ‘true’ *C. nivalis* [4,41]. The latter has tear drop-shaped flagellates with a prominent posterior end. However, Robert Chodat´s type locality of *C. nivalis* in France close to Mont Blanc has not yet been resurveyed for revealing the molecular identity of its population. Moreover, in the original description the flagellate morphology is missing [66]. Recent molecular data suggest that species with a *C. nivalis*-like morphology of cysts evolved several times in the history of snow-thriving *Chloromonas* species [4,52,61].

### 4.5. Photobiology of Field Cysts vs. Laboratorial Strain

The rapid light curves from different habitats showed a high intraspecific flexibility of *C. hindakii* in terms of light acclimation, reflecting the occurrence in semi-shaded locations close to spruce canopy, below dwarf pine vegetation, as well as in exposed sites above timberline. This likely indicates the capabilities of the photosynthetic apparatus in regard to changeable short- and long-term incident irradiations, such as (a) diurnal changes due to topographic or/and vegetation shadings [48], (b) the distance of the cells from the snow surface during melting season [16], and (c) actual weather situation (e.g., sunny vs. cloudy day; [67]).

The fluorometric measurements of cysts showed that the photosystem II was well adapted to medium-to-high levels of irradiation. The cysts from the high light conditions (red graph in Figure 8) became photoinhibited at much higher irradiances than cysts from low light conditions (blue and orange graphs in Figure 8) or the laboratory strain, which was grown at low-light conditions (green graph in Figure 8). At open sites, at a snow depth of 20 cm, usually less than 5% of incident irradiation may be available [15]. Thus, in case of this study, the cysts found at an alpine location 5 cm below snow surface (sample LP06) perceived similarly low PAR like the cells at snow surface below spruce canopy (sample DD2). As a consequence, the characteristics of the rapid light curves (i.e., alpha, I_k_ and ETR values) of cells from these two different shaded sites are similar, which indicates that acclimation of the photosynthesis to certain snow depths is similar for *C. hindakii* with acclimation to a snow surface at semi-shaded site. Semi-shaded sites below timberline differ from open sites above timberline in the amplitude of PAR; for the former in the Krkonoše Mountains it reaches up to ~1300 µmol photons m^2^ s^−1^ [48]. In contrast, the cysts from open alpine sites (sample WP194) in the High Tatra Mountains were exposed to PAR exceeding 2000 µmol photon m^2^ s^−1^ (see Supplementary Figure S27 in [16]). Likewise, other species were still able to perform positively at such high irradiances, and the light saturation (optimal) irradiance (i.e., I_k_) of *C. hindakii* was nearly as high as those for cysts of *C. nivalis* and *C. nivalis* subsp. *tatrae,* causing reddish/brownish snow [18,20]. In contrast, the strain of *C. hindakii* exhibited a similarly low I_k_ and high light utilization efficiency (alpha value) to those recorded for cysts of the snow alga *Scotiella cryophila* caused green subsurface stripes at a depth of 20 cm below the snow surface [22]. In other words, photosynthesis was adapted to low light conditions. The high intraspecific flexibility of *C. hindakii*’s response to different light conditions allows this species to colonize, respectively survive at changeable conditions in snow.

### 4.6. Pigments and Fatty Acid Composition

The average levels of astaxanthin that cause the reddish coloration of *C. hindakii* are comparable to mature cysts of *C. nivalis* from the Austrian Alps [18]. Still, the astaxanthin to chlorophyll *a* ratio is three times lower than for *C. nivalis* subsp. *tatrae* from exposed sites at the High Tatras [20]. The main role of astaxanthin is likely to act as sunscreen against excessive visible and harmful UV irradiation. On the other hand, too much astaxanthin would shade the plastids too much, thus decreasing the photosynthetic performance at sites with lower irradiation levels. Apparently, this species has developed a compromise. Probably, the accumulation of astaxanthin reflects the extent of endogenous cyst maturation, regardless of whether the cells are found at high light or at low light conditions (i.e., WP194 had similar astaxanthin to chl-*a* ratio like LP06). The laboratorial strain of *C. hindakii* produces no astaxanthin (data not shown), different to other microalgae feasible for biotechnology [68].

With the same protocol as for pigments, the plastid antioxidant α-tocopherol was quantified. The cysts of *C. hindakii* showed one magnitude higher concentrations than *C. nivalis* from the Austrian Alps [18], but the quantities were similarly low to cells that cause red snow (*Sanguina nivaloides*, referred as *Chlamydomonas nivalis* in [23]). The accumulation of vitamin E was reported to increase during the maturation process of *Chlainomonas* sp. cysts under harsh environmental conditions in the late melting season [16].

In both stages, the strain and the field cysts of *C. hindakii* had a high level of PUFAs, which is regarded as an adaptation to cold conditions [69]. High levels of PUFAs are common also for other snow-dwelling *Chloromonas* species (e.g., see FAs composition in Table 5 in [20]) and algae living in other cold habitats such as lake ice [70]. Hexadecatetraenoic acid was present in flagellates of *C. hindakii* at relatively high levels of total FAs (~30%); its production in *C. hindakii* was the same as in a strain of *C. remiasii* CCCryo 005-99 isolated from snow in the Arctic and kept under nitrogen deficient conditions [71]. A likewise high accumulation of hexadecatetraenoic acid was found in edible marine macroalgae *Undularia pinnatifida* and *Ulva pertusa* [72]. This fatty acid extracted from algae was shown to have physiological effects on the human health [73]; it inhibited production of leukotrienes involved in several inflammatory and allergic reactions (e.g., [74]). What is more, alpine and polar microalgal strains are promising candidates for biotechnology [75], also in outdoor bioreactors at low temperature conditions [76]. Higher levels of saturated palmitic acid and monounsaturated oleic acid in *C. hindakii* correspond to similar amounts found in other strains of genus *Chloromonas* [77].

## 5. Conclusions

Molecular phylogenetic assays, in combination with morphological observation of cysts (likely zygotes) and vegetative flagellates, demonstrated that *C. hindakii* is a new species within a group of morphologically and taxonomically closely related cryoflora phytoflagellates, and most of them were formerly associated with the collective taxon *C. nivalis*. An unialgal vegetative strain with motile flagellates was established from field-collected cysts under suitable laboratorial conditions simulating the temperature of melting snow. For further detail in physiological, cytological, or closer molecular investigations, a clonal strain should be derived from a single cell. *C. hindakii* occurred at very different altitudes from the montane belt to subalpine and alpine locations of mid-latitude mountains, and it exhibited an infraspecific photosynthetic flexibility accordingly. This study shows for the first time for a snow alga that populations from high light conditions get photoinhibited at higher irradiances than those harvested from low light conditions. The physiologic light preferences reflected the light conditions in the original habitat. PUFAs prevailed in the lipids of *C. hindakii*, and astaxanthin was the pigment responsible for the orange coloration of the snow bloom. Generally, the response of a cold-adapted alga exposed to different light conditions can be investigated, along with screening which responses are activated (e.g., effect on pigments, proteins), as done for *Koliella antarctica* [78]. In future, the intraspecific variability among populations of other snow algae and its relation to the incident irradiation may be tested.

## Figures and Tables

**Table 1:** Sampling locations of *Chloromonas hindakii* sp. nov. from Krkonoše, Jeseníky, and High (H.) Tatra Mountains with sample codes, collection date, sampling site, altitude (m a.s.l.) and geographic position (GPS) (CZ, Czech Republic; PL, Poland; SK, Slovakia). The numbers in parentheses indicate position in the map of Figure 1 (L., Lake).

Sample	Date	Location	Altitude	GPS
DD2	11.5.2017	CZ (1), Krkonoše, Dlouhý důl	984	N50°43.231 E15°39.433
Rozcestí	12.5.2017	CZ (2), Krkonoše, close to Na Rozcestí	1349	N50°42.327 E15°40.368
Jes19-1	8.5.2019	CZ (3), Jeseníky, close to U Výrovky	1137	N50°06.673 E17°10.967
Jes19-6	9.5.2019	CZ (4), Jeseníky, next to Sněžná kotlina	1157	N50°08.121 E17°08.817
LP06	14.6.2017	PL (5), H. Tatras, Dolina za Mnichem	1858	N49°11.656 E20°03.146
WP129	15.6.2017	PL (6), H. Tatras, Dolina za Mnichem	2082	N49°11.424 E20°03.153
WP130	15.6.2017	PL (7), H.Tatras, ice-covered Zadni Mnichowy L.	2038	N49°11.403 E20°03.101
WP136	15.6.2017	SK (8), H. Tatras, Mengusovská dolina	1976	N49°10.475 E20°04.910
NW	18.6.2017	SK (9), H. Tatras, shore of Nižné Wahlenberg.L.	2061	N49°9.543 E20°1.612
WP194	17.6.2018	SK (10), H. Tatras, Velká Studená dolina	2022	N49°10.540 E20°09.414

**Table 2:** List of primers used for amplification of 18S rDNA, ITS1 rDNA, ITS2 rDNA (ITS), and *rbc*L markers (F, forward; R, reverse).

Primer	Marker	Direction	Sequence	Reference
SSU	ITS2	F	CTGCGGAAGGATCATTGATTC	[26]
LSU	ITS2	R	AGTTCAGCGGGTGGTCTTG	[26]
ITS5	ITS2	F	GGAAGTAAAAGTCGTAACAAGG	[27]
ITS1	ITS2	F	TCCGTAGGTGAACCTGCGG	[27]
ITS4	ITS2	R	TCCTCCGCTTATTGATATGC	[27]
Al1500af	ITS2	F	GCGCGCTACACTGATGC	[28]
LR3	ITS2	R	GGTCCGTGTTTCAAGACGG	[29]
18F2	18S	F	AACCTGGTTGATCCTGCCAGT	[30]
18R2	18S	R	TGATCCTTCTGCAGGTTCACCTACG	[30]
rbcL1F	*rbc*L	F	CTGCTTTATACTGCGAAACTGC	[31]
rbcL7R	*rbc*L	R	AAATAAATACCACGGCTACG	[31]

**Table 3:** List of marker sequences for the authentic strain of *Chloromonas hindakii* and field samples, indicating the Genbank accession numbers for ITS2 rDNA/18S rDNA/*rbc*L sequences.

Sample/Strain Code	NCBI Accession Numbers		
	ITS2 rDNA	18S rDNA	*rbc*L
WP129 (= CCCryo 531-19)	MN251865	MN251865	MN251877
DD2	MN251866	MN251874	MN251878
Rozcestí	MN251867	MN251875	MN251879
Jes19-1	MN251868		
Jes19-6	MN251869	MN251876	
LP06	MN251870		
WP136	MN251871		
NW	MN251872		MN251880
WP194	MN251873		MN251881

**Table 4:** Abiotic habitat parameters and cell sizes of *Chloromonas hindakii* field samples from the Krkonoše, Jeseníky, and High Tatra Mountains. Electrical conductivity (EC; μS.cm^−1^), pH of meltwater and snow water content (SWE; %), maximal population density ± standard deviation (SD), average sizes of cells in μm ± SD, length to width ratio (L:W ratio) ± SD are shown.

Sample	EC	pH	SWE	Cells Per mL Meltwater	Cell Length	Cell Width	L:W Ratio
DD2	14	5.7	48.7 ± 0.3	19,950 ± 2000	25.5 ± 2	16.3 ± 1.4	1.57 ± 0.1
Rozcestí	9	5.9	-	-	25.0 ± 2.1	16.6 ± 1.5	1.51 ± 0.1
Jes19-1	28	7.0	-	60,800 ± 6080	27.3 ± 2.1	17.9 ± 2.2	1.53 ± 0.1
Jes19-6	33	6.9	-	43,050 ± 4300	30.3 ± 2.1	19.7 ± 1.7	1.54 ± 0.1
LP06	5.2	5.5	55.9 ± 2.1	54,150 ± 5400	25.2 ± 2	16.4 ± 1.5	1.54 ± 0.1
WP129	-	-	-	-	23.7 ± 1.2	15.0 ± 0.8	1.52 ± 0.1
WP130	-	-	-	47,100 ± 4700	-	-	-
WP136	-	-	-	79,100 ± 7900	23.5 ± 2.3	15.6 ± 2.1	1.52 ± 0.1
NW	-	-	-	-	23.0 ± 2.1	15.0 ±1.4	1.54 ± 0.1
WP194	5.1	6.8	60.8 ± 5.97	21,900 ± 2100	26.8 ± 1.4	17.6 ± 1.4	1.53 ± 0.1

**Table 5:** Relative cellular content of carotenoids, chlorophyll *b,* and α-tocopherol in relation to chlorophyll *a* (= 1) in field samples of *Chloromonas hindakii* sp. nov. from the High Tatras at the Slovak side (sample WP194; high light conditions) and at the Polish side (LP06; low light conditions), determined by HPLC. Abbreviations: n & v, neoxanthin and violaxanthin; lut, lutein; zea, zeaxanthin; chl *b*, chlorophyll *b*; β-car, β-carotene; ast, astaxanthin (free/unesterified); ast-E, astaxanthin derivatives (esters); ast-tot, astaxanthin in total (free and derivatives); α-toc; α-tocopherol; n.d., not detected.

Sample	N&V	Lut	Zea	Chl *b*	β-car	Ast	Ast-E	Ast-tot	α-toc
WP194	0.120	0.315	n.d.	0.227	0.014	0.037	0.385	0.422	0.085
LP06	0.135	0.277	n.d.	0.273	0.020	0.012	0.476	0.488	0.080

**Table 6:** Cellular fatty acid composition of *Chloromonas hindakii* sp. nov. vegetative strain WP129 (= CCCryo 531-19) cultivated at 1 °C (*n* = 3) compared to field-collected cysts (from sample Rozcestí) in % of total lipids (TL) and in % of the three major lipid groups: Neutral lipids (NL), phospholipids (PL), and glycolipids (GL). The table shows only fatty acids that have abundances greater than 0.1%. The relative proportion of saturated (SAFA), monounsaturated (MUFA), and polyunsaturated (PUFA) fatty acids is also given.

Fatty Acid	WP129 (= CCCryo 531-19)				Rozcestí
	TL	NL	PL	GL	TL
14:0	0.7 ± 0.5	0.8 ± 0.6	1.1 ± 0.3	0	0.5
16:0	14.7 ± 6.1	15.6 ± 8.3	15.7 ± 1.4	10.4 ± 0.9	19.2
16:1 (9Z)	0.3 ± 0.1	0	2.5 ± 1.3	0	1.9
16:1 (7Z)	0.4 ± 0.3	0.5 ± 0.5	0	0	2.1
3t-16:1	0	0	4.6 ± 0.1	0	0
16:2 (7Z,10Z)	0	0	0	0	3.2
16:3 (4Z,7Z,10Z)	0	0	0	0	0.3
16:3 (7Z,10Z,13Z)	2.1 ± 1.1	1.9 ± 1.4	1.5 ± 0.2	3.6 ± 0.3	2.7
16:4 (4Z,7Z,11Z,13Z)	28.5 ± 4.6	30.6 ± 6.1	12.1 ± 0.6	29.5 ± 1.6	10.3
18:0	7.3 ± 6.8	6.7 ± 8.9	1.5 ± 0.6	14.5 ± 3.6	1.3
18:1 (11Z)	0.5 ± 0.3	0.5 ± 0.4	1.1 ± 0.3	0	7.5
18:1 (9Z)	9.8 ± 3.6	12.1 ± 5.2	8.4 ± 1.1	0	9.4
18:2 (9Z,12Z)	3.4 ± 0.8	3.9 ± 1.0	3.4 ± 0.1	1.2 ± 0.7	4.8
18:3 (9Z,12Z,15Z)	25.9 ± 3.7	21.4 ± 3.7	44.7 ± 1.7	33.2 ± 8.4	31.6
18:3 (6Z,9Z,12Z)	0	0	0	0	0.5
18:4 (6Z,9Z,12Z,15Z)	5.9 ± 0.2	6.0 ± 1.6	3.3 ± 1.1	7.4 ± 4.7	4.7
SAFA	22.7 ± 10.9	23.1 ± 13.5	18.4 ± 1.0	25.0 ± 3.6	21
MUFA	11.5 ± 3.6	13.1 ± 5.0	16.6 ± 0.8	0	20.9
PUFA	65.8 ± 9.9	63.8 ± 8.5	65.1 ± 1.5	75.0 ± 3.6	58.1

## Supplementary Materials

The following are available online at https://www.mdpi.com/2076-2607/7/10/434/s1
