# Supplementary material for: Ecophysiology of Chloromonas hindakii sp. nov. (Chlorophyceae), Causing Orange Snow Blooms at Different Light Conditions
Source: Microorganisms. 2019 Oct 10;7(10):434. doi: 10.3390/microorganisms7100434 (PMC6843554; doi:10.3390/microorganisms7100434)
Supplement: Supplementary file 1 [file microorganisms-07-00434-s001.pdf]

Supplementary Information

Ecophysiology of *Chloromonas hindakii* sp. nov. (Chlorophyceae), Causing Orange Snow Blooms at Different Light Conditions

Lenka Procházková, Daniel Remias, Tomáš Řezanka and Linda Nedbalová

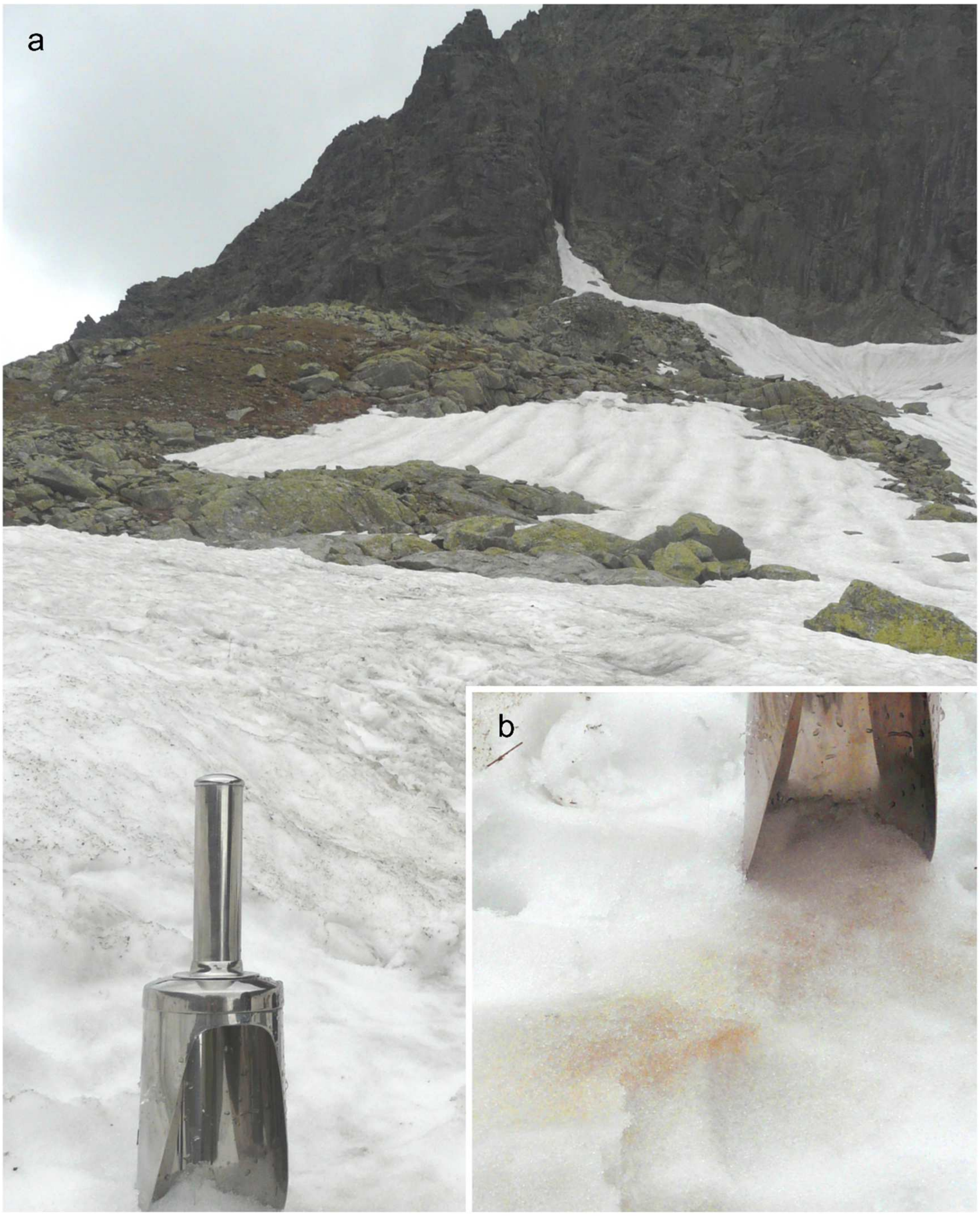

Figure S1. Type locality of *Chloromonas hindakii* sp. nov. at Dolina za Mnichem, Powiat tatrzański, Bukowina Tatrzańska, High Tatra Mountains, Poland: (**a**) overview of the sampling location, (**b**) detail view of the orange snow spot during harvest (sample WP129, June 2017).

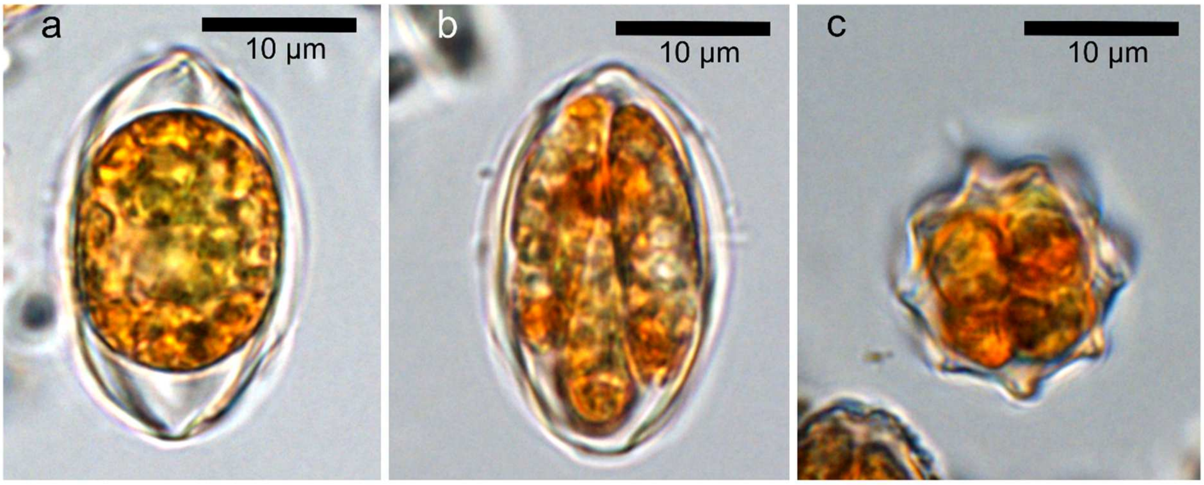

Figure S2. Light microscopy of field-collected material of *Chloromonas hindakii* sp. nov. (sample WP129). The cysts were kept in deionized water for several weeks at temperatures around the freezing point to provoke cell division (putative meiosis). (**a**) Old cyst, preparing for meiosis (or mitosis) by contracting the protoplast. In a consecutive step, (**b**) four elongated cells developed, still surrounded by the mother cell wall, lateral view, (**c**) apical view of the smooth walled daughter cells.

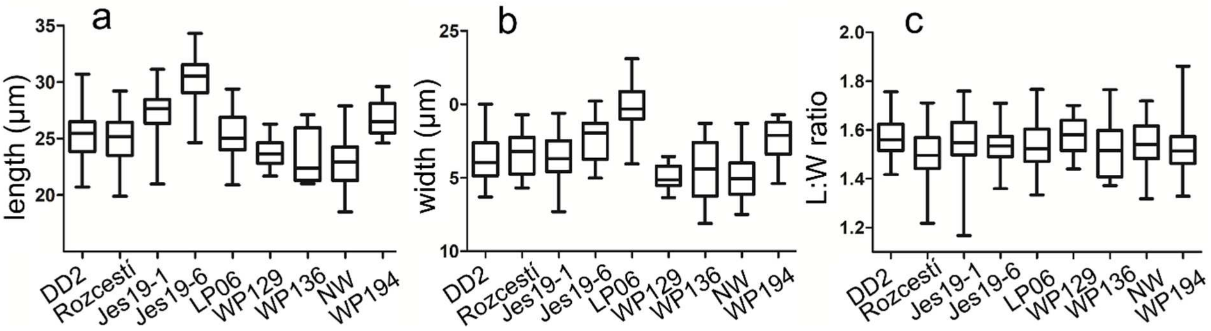

Figure S3. Cell size ranges of field-collected cysts of *Chloromonas hindakii* sp. nov. from the Krkonoše Mountains, the High Tatras and the Jeseníky Mountains after harvest (DD2, n = 40; Rozcestí, n = 32; LP06, n = 44; WP129, n = 21; WP136, n = 9; NW = 38; WP194, n = 31; Jes19-1, n = 33; Jes19-6, n = 36): (**a**) length, (**b**) width, (**c**) length to width ratio.

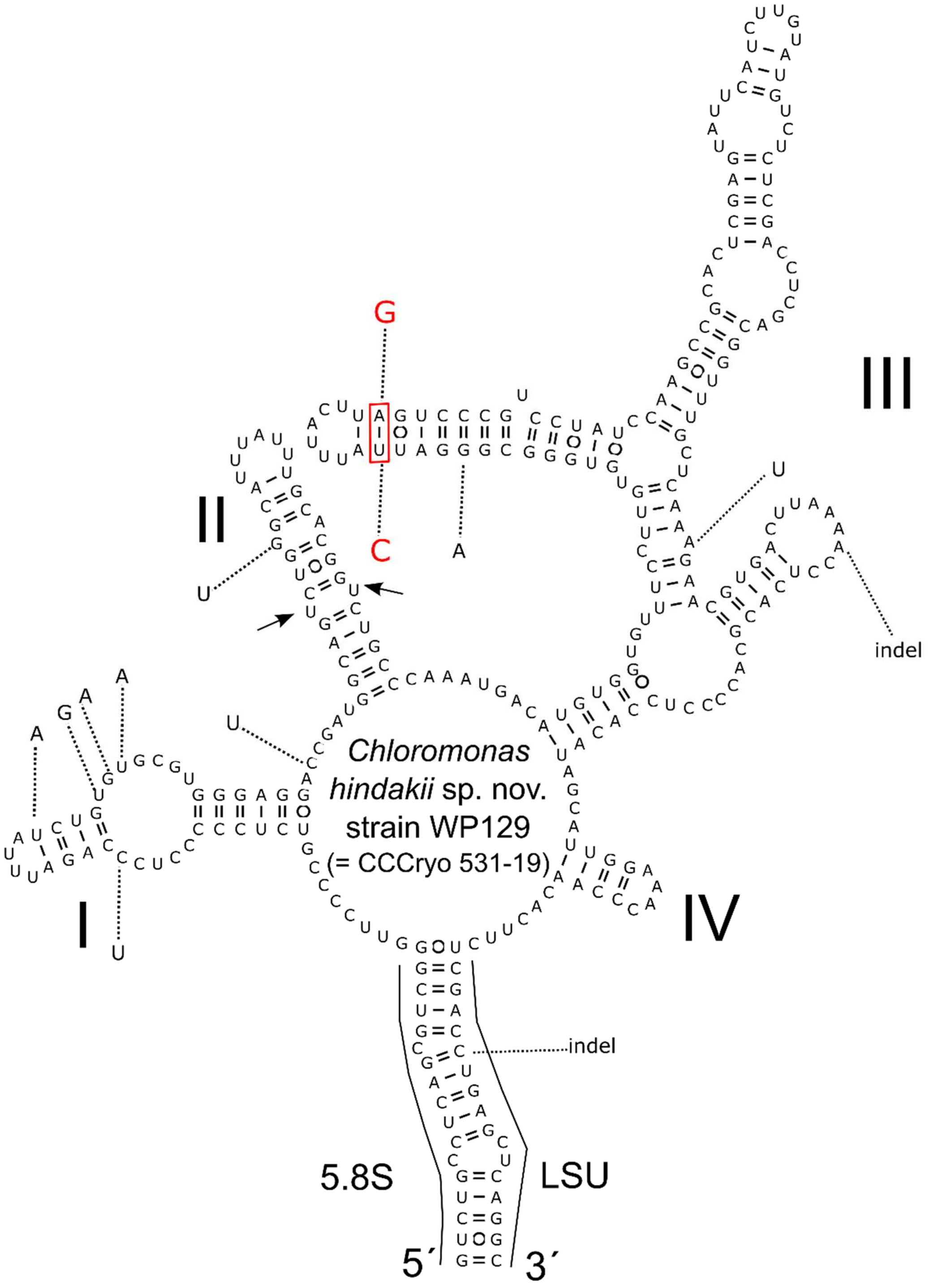

Figure S4. Comparison of the secondary structure of ITS2 rDNA transcripts between *Chloromonas hindakii* sp. nov. strain WP129 (= CCCryo 531-19, accession number MN251865) and the Uncultured Chlorophyte clone ALBC6 from Swiss Alps (accession number JX435348; [51]). Helices are labelled with Latin numbers: I–IV. Nucleotide differences of the second species are described in red outside the structure and linked by dotted lines. Compensatory base change in the 5′end of helix III between both algae is indicated by a rectangle. Note the U–U mismatch in helix II (arrows).

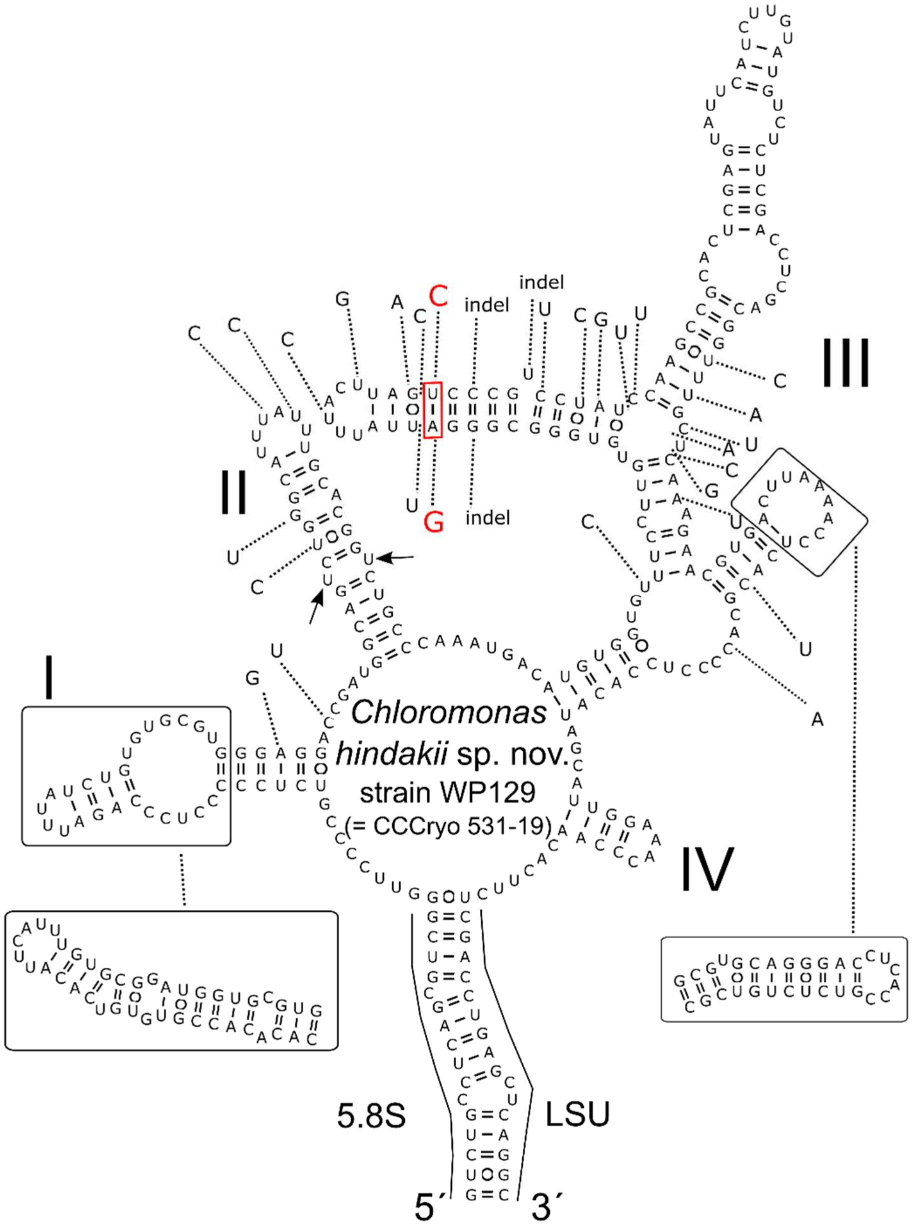

Figure S5. Comparison of the secondary structure of ITS2 rDNA transcripts between *Chloromonas hindakii* sp. nov. strain WP129 (= CCCryo 531-19, accession number MN251865) and *Chloromonas nivalis* Gassan-B from Japan (accession number LC012758, [52]). Helices are labelled with Latin numbers: I–IV. Nucleotide differences of the second species are described in red outside the structure and linked by dotted lines. Compensatory base change in the 5´end of helix III between both algae is indicated by a rectangle. Note the U–U mismatch in helix II (arrows).

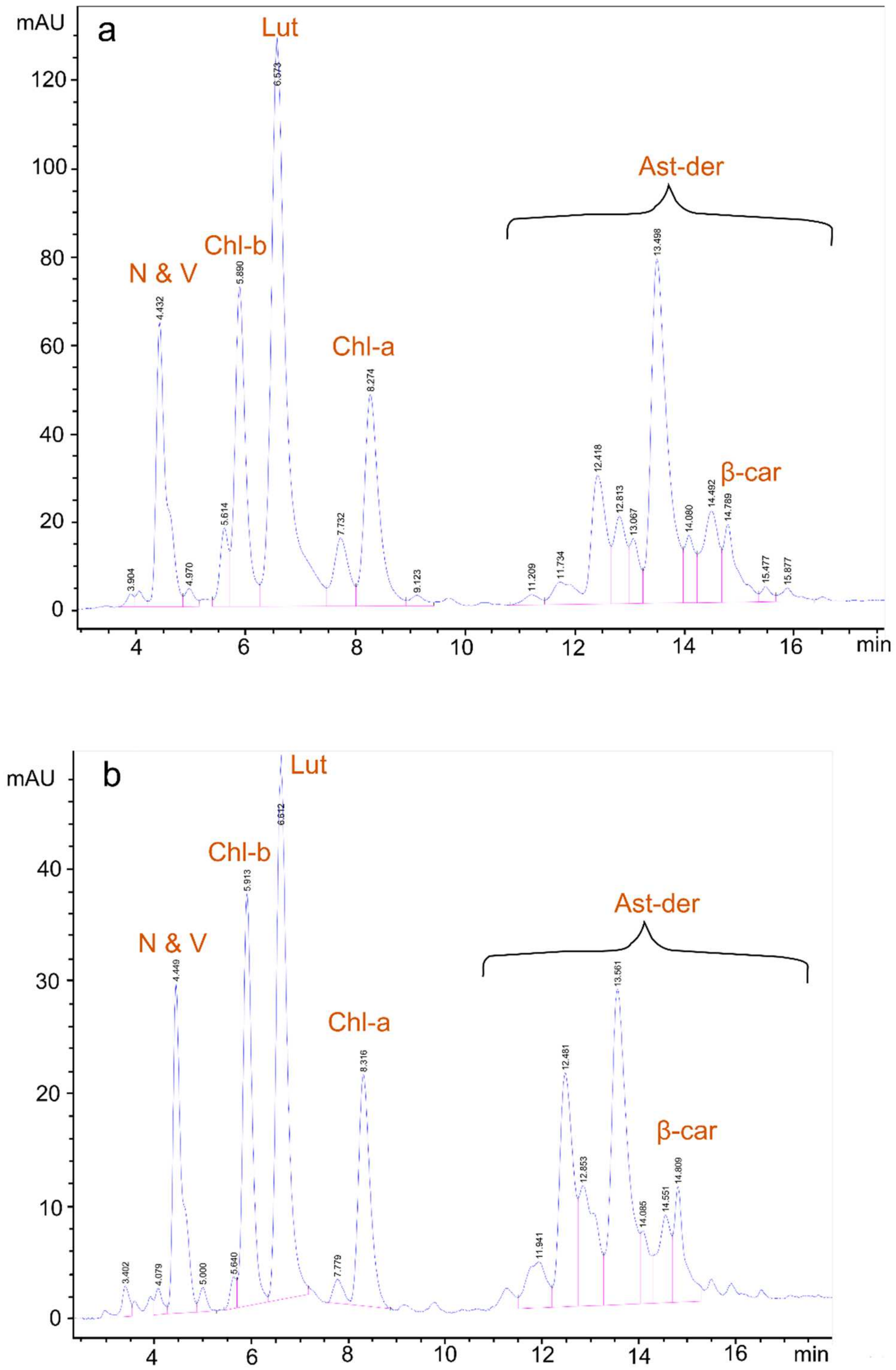

Figure S6. HPL-chromatogram of field-collected cysts of *Chloromonas hindakii* sp. nov. at 450 nm showing the main pigments. (**a**) Sample WP194 (high light conditions) from the Slovak side of the High Tatras. (**b**) Sample LP06 (low light conditions) from the Polish side of the High Tatras. Abbreviations: N, neoxanthin; V, violaxanthin; Lut, lutein; Chl-b, chlorophyll b; Chl-a, chlorophyll a; β-car, β-carotene; Ast-der, Astaxanthin derivatives (pooled).
